# Supplementary material for: Empirical methods for controlling false positives and estimating confidence in ChIP-Seq peaks
Source: BMC Bioinformatics. 2008 Dec 5;9:523. doi: 10.1186/1471-2105-9-523 (PMC2628906; doi:10.1186/1471-2105-9-523)
Supplement: Additional File 1 — A variety of html documents from the USeq web site detailing the available applications, their best usage, output file type descriptions, command line menus, etc. [file 1471-2105-9-523-S1.zip › USeqUserGuides/JavaDoc.zip!/JavaDoc/trans/roc/class-use/Sgr.html]

Uses of Class trans.roc.Sgr


|  |  |  |
| --- | --- | --- |
| **Overview** \| **Package** \| **Class** \| **Use** \| **Tree** \| **Deprecated** \| **Index** \| **Help** | |  |
| PREV   NEXT | **FRAMES**    **NO FRAMES**     **All Classes** |  |


---


## **Uses of Class trans.roc.Sgr**

| Packages that use Sgr | |
| --- | --- |
| **trans.graphics** | For graphically representing '.cel' files and intervals. |
| **trans.misc** | Rough working scripts. |
| **trans.roc** | For manipulating the BAC dmel spike in data and generating ROC curves. |

Uses of Sgr in trans.graphics

| Methods in trans.graphics with parameters of type Sgr | |
| --- | --- |
| `static void` | `AggregatePlots.loadRegions(Sgr sgr, Positive[] pos)`             Checks to see if an sgr object is in the positives, if so, adds the sgr object to the positive. |

Uses of Sgr in trans.misc

| Methods in trans.misc that return Sgr | |
| --- | --- |
| `static Sgr[]` | `Util.loadGrFile(java.io.File grFile, java.lang.String chromName)`             Reads in an sgr file. |
| `static Sgr[]` | `Util.loadSgrFile(java.io.File sgrFile)`             Reads in an sgr file. |

Uses of Sgr in trans.roc

| Methods in trans.roc that return Sgr | |
| --- | --- |
| `static Sgr[]` | `MethodTester.parseSgrFile(java.io.File sgrFile, boolean zeroNaNOrInfinityScores)`             Reads in an Sgr file. |
| `static Sgr[][]` | `Sgr.splitByChromosome(Sgr[] sortedSgrs)`             Splits a Sgr[] containing many chromosome lines to a Sgr[chrom][lines]. |

| Methods in trans.roc that return types with arguments of type Sgr | |
| --- | --- |
| `static java.util.ArrayList<Sgr>` | `Sgr.loadSgrFile(java.io.File sgrFile)`             Loads an xxx.sgr(.zip) file into an ArrayList of Sgr |

| Methods in trans.roc with parameters of type Sgr | |
| --- | --- |
| `Bin` | `SgrBacCounter.findBin(Sgr sgr)`             Finds an appropriate bin given an sgr object |
| `Bin` | `MethodTester.findBin(Sgr sgr)`             Finds an appropriate bin given an sgr object |
| `static int` | `ScoreParsedBars.inRegion(Sgr sgr, Positive[] pos)`             Checks to see if an sgr object is in one of the positives, if not returns -1, otherwise returns index number. |
| `static int` | `ParseSgrsForParticularRegions.inRegion(Sgr sgr, Positive[] pos)`             Checks to see if an sgr object is in one of the positives, if not returns -1, otherwise returns index number. |
| `static void` | `ParseSgrsForParticularRegions.loadRegions(Sgr sgr, Positive[] pos)`             Checks to see if an sgr object is in the positives, if so, adds the sgr object to the positive. |
| `boolean` | `Positive.matches(Sgr sgr)` |
| `static int` | `SgrBacCounter.positive(Positive[] pos, Sgr sgr)`             Checks to see if an sgr object is one of the positives, if not returns -1, otherwise returns index number. |
| `static int` | `MethodTester.positive(Positive[] pos, Sgr sgr)`             Checks to see if an sgr object is one of the positives, if not returns -1, otherwise returns index number. |
| `static GrGraph[]` | `Sgr.splitByChrom(Sgr[] sortedSgrs)`             Splits a Sgr[] by chromosome. |
| `static Sgr[][]` | `Sgr.splitByChromosome(Sgr[] sortedSgrs)`             Splits a Sgr[] containing many chromosome lines to a Sgr[chrom][lines]. |

---


|  |  |  |
| --- | --- | --- |
| **Overview** \| **Package** \| **Class** \| **Use** \| **Tree** \| **Deprecated** \| **Index** \| **Help** | |  |
| PREV   NEXT | **FRAMES**    **NO FRAMES**     **All Classes** |  |


---
